# Parotid neoplasms: analysis of 600 patients attended at a single institution

**DOI:** 10.1016/S1808-8694(15)30486-9

**Published:** 2015-10-19

**Authors:** Ademar Takahama, Oslei Paes de Almeida, Luiz Paulo Kowalski

**Affiliations:** 1 Master's degree, doctoral student in the graduate stomatopathology program at the Faculdade de Odontologia de Piracicaba – Universidade Estadual de Campinas; 2 Doctorate, full professor of the oral diagnosis department, Faculdade de Odontologia de Piracicaba – Universidade Estadual de Campinas; 3 Doctorate, head of the otorhinolaryngology and head & neck surgery department, Hospital A. C. Camargo, Sao Paulo. Oral Diagnosis Department. Faculdade de Odontologia de Piracicaba. Universidade Estadual de Campinas. Otorhinolaryngology and Head & Neck Surgery Department, Hospital A.C. Camargo, São Paulo

**Keywords:** pleomorphic adenoma, mucoepidermoid carcinoma, parotid gland, neoplasm

## Abstract

Salivary gland tumors are rare, generally benign and affect mainly the parotid gland. **Aim.** The purpose of this study was to retrospectively analyze all cases of parotid tumors treated at our institution from 1953 to 2003. **Methods.** All patients with primary parotid tumors were selected; clinical and histopathological data were analyzed and described. **Results.** 600 cases of parotid tumors were selected; 369 were benign and 231 were malignant. Pleomorphic adenoma was the most frequent benign tumor. The most common malignant tumor was the mucoepidermoid carcinoma. Therapy in most cases consisted of parotidectomy. Adjuvant therapy – mainly radiotherapy – was used in some cases with malignant tumors. The incidences of local, regional and distant recurrences of malignant tumors were 10%, 8% and 9%. **Conclusion.** Patients with parotid tumors treated at our institution were mainly adults, with marginally more female patients. Benign tumors were mostly the pleomorphic adenoma, which were more frequent than malignancies. Most of the patients were treated by partial or total parotidectomy. Adjuvant therapy, mainly radiotherapy, was used in selected malignant cases.

## INTRODUCTION

Salivary gland neoplasms are a rare group of tumors; the annual incidence rate is 1 in 100,000 individual, comprising about 3% of all head and neck neoplasms.^1^ The mean age of patients with salivary gland tumors is 45 years, peaking in the sixth and seventh decades of life.^2^, ^3^ Benign salivary gland tumors occur more frequently in females, while malignant tumors are slightly more frequent in males.^4^,^5^

The parotid gland is the most frequent site – about 70% of cases.^4^, ^5^, ^6^ About 80% of parotid tumors are benign, the most common being the pleomorphic adenoma (60% of parotid tumors), followed by Warthin's tumor (10% of parotid tumors).^4^

The most common salivary gland malignancy is the mucoepidermoid carcinoma, which involves mostly the parotid gland, followed by the minor, submandibulary and sublingual salivary glands.^5^, ^6^, ^7^ The cystic adenoid carcinoma is the second most frequent malignancy in this area; other parotid tumors are acinar cell carcinomas, adenocarcinoma NOS, and the carcinoma ex pleomorphic adenoma.^1^

The main symptom in patients with parotid neoplasms is a lump in the parotid area. Other symptoms such as pain, facial palsy and skin ulcers may manifest in malignant cases.^8^,^9^ The treatment of choice for benign and malignant parotid tumors is partial or total parotidectomy, according to the extent of the tumor.10 Radiotherapy may be useful in malignancies as adjuvant therapy; chemotherapy is rarely used.^6^, ^11^, ^12^ Local, regional and distance recurrence rates are 40%, 15% and 11% each, and worsened the prognosis.^6^ The purpose of this study was to assess the clinical and histological features of all patients with primary parotid tumors treated at a single institution during a 50-year period.

## SERIES AND METHOD

The Research Ethics Committee approved this study (protocol – 711/05). All primary parotid tumor cases treated at our institution from 1953 to 2003 were selected for this study. Cases were excluded when the patient file was not located, when information was lacking in the file, when paraffin blocks and slides were not available, or when patients were treated at another institution. Clinical data were gathered from medical files. A histological review of all cases was done based on the World Health Organization classification.^13^

## RESULTS

The study sample consisted of 600 cases with the necessary information for analysis. There were 369 benign cases (60%) and 231 malignancies (40%). The mean age was 48.4 years. There were slightly more females in the sample (53%).

### Benign

The most frequent benign tumors were the pleomorphic adenoma (66,5%) and Warthin's tumor (25%). Other less frequent tumors were the basal cell adenoma, the oncocytoma and the myoepithelioma. Fifteen cases presented mesenchymal tumors: five lymphangiomas, 5 neurofibromas, and 1 case each of lipoma, schwanoma, solitary fibrous tumor, giant cell tumor and meningioma (Table 1).

**Table 1:** Distribution of 369 benign tumors of the parotid gland treated at our institution, according to the histological type.

Histological type	Number of cases	Percentage
Pleomorphic adenoma	245	66,5%
Warthin's tumor	93	25%
Basal cell adenoma	10	3%
Oncocytoma	4	1%
Myoepithelioma	2	0,5%
Mesenchymal tumors	15	4%
TOTAL	369	100%

The mean of patients with benign tumors was 47 years; the peak incidence was the fifth decade of life. There were slightly more female patients – 55% of cases. In pleomorphic adenomas females comprised 62% of cases; in Warthin's tumor, 65.5% of cases were males. The mean duration of complaints was 40 months; the main symptoms were lumps in the parotid area (98% of cases) and pain (11% of cases). The mean size of tumors was 4 cm (ranging from 1 to 30 cm). Ten patients presented bilateral tumors (both parotid glands), all of which were diagnosed as Warthin's tumor. All patients underwent surgery; 330 cases (90%) underwent partial parotidectomy and 38 cases (10%) underwent total parotidectomy. The facial nerve was fully preserved in 365 cases; at least one of its branches was sacrificed in 3 cases. The main postoperative complication was Frey's syndrome (34 cases – 9%). Facial palsy was present in 34 cases; it was temporary in 28 cases and permanent in 6 cases. Local recurrence occurred in ten cases after 18 to 112 months (mean – 56 months), all of which with the pleomorphic adenoma.

### Malignant

The most common malignancy was the mucoepidermoid carcinoma, followed by the undifferentiated carcinoma, the cystic adenoid carcinoma, the adenocarcinoma NOS, the acinar cell carcinoma, the carcinoma ex pleomorphic adenoma, and the squamous cell carcinoma (Table 2). There were 24 non-epithelial origin malignancies, of which 14 were lymphomas. The mean age of patients with malignant tumors was 50 years, peaking at the sixth decade of life. Males comprised 52% of cases. The mean duration of complaints was 35 months; the main symptom was a lump in the parotid area (220 cases – 91%). Pain was reported in 30% of cases, facial palsy in 10% of cases, and trismus in 6% of cases. The mean diameter of tumors was 5.5 cm (ranging from 1 to 20 cm).

**Table 2:** Distribution of 231 malignant tumors of the parotid gland treated at our institutions, according to the histological type.

Histological type	Number of cases	Percentage
Mucoepidermoid carcinoma	67	29%
Undifferentiated carcinoma	33	13,5%
Cystic adenoid carcinoma	27	11%
Adenonocarcinoma NOS	22	9%
Acinar cell carcinoma	20	8%
Carcinoma ex pleomorphic adenoma	19	8%
Squamous cell carcinoma	15	6%
Basal cell adenocarcinoma	5	3%
Salivary duct carcinoma	4	2%
Lymphoepithelial carcinoma	2	1%
Epithelial-Myoepithelial carcinoma	1	0,5%
Oncocytic carcinoma	1	0,5%
Myoepithelial carcinoma	1	0,5%
Lymphoma	14	6%
Non-lymphoid mesenchymal tumor	4	2%
TOTAL	231	100%

TNM staging was as follows: 23 cases were T1, 80 cases were T2, 59 cases were T3, 70 cases were T4, and the primary tumor was not classified in 9 cases. Palpable lymph nodes were found in 39 cases: N1 in 21 cases, N2 in 5 cases, and N3 in 10 cases. Three cases were classified as M1, all with lung metastases.

The main form of therapy was surgery; partial parotidectomy was done in 36.3% of cases, total parotidectomy was done in 57% of cases, and extended parotidectomy was done in 6.7% of cases (including resection of adjacent structures such as muscle, mandible, ear and skin. The facial nerve was preserved in 72% of cases; it was partially resected in 9% of cases, and sacrificed in 19% of cases. Neck dissection, as well as parotidectomy, was done in 73 cases; metastatic lymph nodes were confirmed histopathologically in 38 cases. Postoperative radiotherapy was carried out in 72 cases; the mean dose was 4,800 Gy. Palliative therapy with radiotherapy only was done in 20 cases; radiotherapy was associated with chemotherapy in 6 cases. Recurrences manifested in 48 patients (21%). Local recurrence occurred in 25 cases (10%); of these, 13 cases were exclusively local recurrences, 4 cases also had regional recurrences, 5 cases had distance metastases, and 2 cases had both regional and distance metastases. Regional recurrence was seen in 19 cases (8%); of these, 10 cases were exclusively regional recurrences, and 3 cases also had distance metastases. Distance metastases were identified in 21 cases (9%), exclusively so in 10 of these cases. The main organ involved in distance metastases was the lung (10 cases).

Our latest information shows that 90 patients died due to disease, 91 were alive and disease-free, 13 were alive with disease, 22 died due to other causes, and 15 were lost to follow-up.

## DISCUSSION

The parotid gland is the main site of salivary gland tumors.^2^,^8^ About 64 to 80% of all primary salivary gland epithelial tumors involve the parotid gland, mostly located in the superficial lobe.^14^ Our sample comprised 600 cases of benign and malignant primary parotid gland tumors.

The incidence of salivary gland tumors peaks in the sixth and seventh decades of life; the mean age was 46 years^3^. Satko et al.^15^ reported a mean age of 53 years (ranging from 2 to 87 years). Most studies show that the mean age is higher in malignant tumors (about 55 years) compared to benign tumors (about 45 years).^2^, ^8^, ^16^ Our results confirm these findings; the mean age in our sample was 48.4 years – 47 years for benign tumors and 50 years for malignancies.

Most of the reviews of salivary gland tumor series show that there are more females than males in both benign and malignant cases.^8^, ^14^, ^17^ This varies depending on the type of tumor; for example, males predominate in Warthin's tumor.^2^ Ito et al.^16^ found that there were more female patients in benign tumors (58.5%), whereas males predominated in malignant tumors (52.2%). We found a slight female predominance (53% of cases), with 55% in benign tumor cases. In malignant tumors, however, males predominated (52% of cases).

The main complaint of patients with parotid tumors was a lump in the parotid area. About 50% parotid malignant tumors had clinical findings that were similar to those of benign tumors, such as slow growth, mobility over underlying tissues, and absence of symptoms. The remaining 50%, however, manifested signs of malignancy, such as facial palsy, pain, trismus, and no mobility.^18^ Most of our benign tumor cases presented a lump in the parotid area. In malignant tumor cases, the parotid volume was increased and 34% of patients also manifested pain, facial palsy and/or trismus.

The pleomorphic adenoma and Warthin's tumor are the most common benign tumors.^3^,^16^ Our data show that the pleomorphic adenoma and Warthin's tumor comprised 66.5% and 25% of cases each. The percentage of Warthin's tumors in our study was high compared to other series of parotid tumors, in which this number generally ranges from 9% to 15%.^8^, ^14^, ^15^, ^19^

Malignant tumors comprise about 15 to 30% of parotid tumors; the most commonly reported of these is the mucoepidermoid carcinoma, followed by the cystic adenoid carcinoma.^1^, ^8^, ^16^ Wahlberg et al.'s^20^ review of 2,062 parotid carcinoma cases revealed that the mucoepidermoid carcinoma was the most common tumor, followed by the adenocarcinoma NOS, the acinar cell carcinoma, the cystic adenoid carcinoma, the carcinoma ex pleomorphic adenoma, and the undifferentiated carcinoma. We also found among our series of malignant tumors that the mucoepidermoid carcinoma was the most common tumor, followed by the undifferentiated carcinoma, the cystic adenoid carcinoma, the adenocarcinoma NOS, the acinar cell carcinoma, and the carcinoma ex pleomorphic adenoma.

The treatment of choice for benign tumors of the parotid gland is parotidectomy, preserving the facial nerve.^21^ Generally, partial parotidectomy is done in tumors limited to the superficial lobe, which is fully resected. Removal of the whole parotid lobe aims to attain adequate surgical margins and avoid rupture of the capsule, which reduces the recurrence rate.^22^ Total parotidectomy in benign tumors is done when the deep lobe of the parotid is involved.^23^ Rupture of the capsule during surgical resection and positive microscopic margins may lead to recurrences.^24^ Parotidectomy was done in all of our cases of benign tumors; only 10 cases had local recurrences. The treatment of choice for parotid malignant tumors is partial or total parotidectomy; the facial nerve is preserved if possible.10 Adjuvant radiotherapy has been shown to be effective for improving local control and increased survival.^12^,^25^ Local, regional and distance recurrence rates for parotid malignant tumors are about 40%, 15% and 11% each; in these cases, the prognosis is worse.6,26 Parotidectomy was done in most of our patients with malignant tumors; adjuvant therapy was used in some cases. Recurrence rates were 10% (local), 8% (regional), and 9% (distance), lower than most of published results in the international literature.

## CONCLUSION

Benign tumors were more frequent in a series of 600 cases of parotid tumors. The pleomorphic adenoma was the most common tumor. The mucoepidermoid carcinoma was the most common malignant tumor. Parotidectomy was the main form of therapy; radiotherapy was reserved for specific malignant tumor cases. Recurrences of any type occurred in 48 patients (21% of cases) during follow-up.

## References

[1] Ellis GL, Auclair PL (1996). Tumors of the salivary glands.

[2] Eveson JW, Cawson RA (1985). Salivary gland tumours. A review of 2410 cases with particular reference to histological types, site, age and sex distribution. J Pathol..

[3] Auclair PL, Ellis GL, Gnepp DR, Wenig BN, Janey CG, Ellis GL, Auclair PL, Gnepp DR (1991). Surgical pathology of the salivary glands.

[4] Nagler RM, Laufer D (1997). Tumors of the major and minor salivary glands: review of 25 years of experience. Anticancer Res..

[5] Pinkston JA, Cole P (1999). Incidence rates of salivary gland tumors: results from a population-based study. Otolaryngol Head Neck Surg..

[6] Yu GY, Ma DQ (1987). Carcinoma of the salivary gland: a clinicopathologic study of 405 cases. Semin Surg Oncol..

[7] Pires FR, Almeida OP, de Araujo VC, Kowalski LP (2004). Prognostic factors in head and neck mucoepidermoid carcinoma. Arch Otolaryngol Head Neck Surg..

[8] Spiro RH (1986). Salivary neoplasms: overview of a 35-year experience with 2,807 patients. Head Neck Surg..

[9] Przewozny T, Stankiewicz C (2004). Neoplasms of the parotid gland in northern Poland, 1991–2000: an epidemiologic study. Eur Arch Otorhinolaringol..

[11] Kessler A, Handler SD (1994). Salivary gland neoplasms in children: a 10-year survey at the Children's Hospital of Philadelphia. Int J Pediatr Otorhinolaryngol..

[12] Bull PD (1999). Salivary gland neoplasia in childhood. Int J Pediatr Otorhinolaryngol..

[13] Eveson JW, Auclair P, Gnepp DR, Barnes L, Eveson JW, Reichart P, Sidransky D (2005). Pathology and genetics of head and neck tumors. World Health Organization Classification of Tumors.

[14] Ellis GL, Auclair PL, Gnepp DR (1991). Surgical Pathology of the Salivary Glands.

[15] Satko I, Stanko P, Longauerova I (2000). Salivary gland tumours treated in the stomatological clinics in Bratislava. J Craniomaxillofac Surg..

[16] Ito FA, Ito K, Vargas PA, Almeida OP, Lopes MA (2005). Salivary gland tumors in a Brazilian population: a retrospective study of 496 cases. Int J Oral Maxillofac Surg..

[17] Williams NP, Boyd DL, Choy L, Hanchard B (2001). Salivary gland lesions: a Jamaican perspective. Wet Indian Med J..

[18] Snow GB (1979). Tumours of the parotid gland. Clin Otolaryngol..

[19] Fitzpatrick PJ, Black KM (1985). Salivary gland tumors. J Otolaryngol..

[20] Wahlberg P, Anderson H, Biörklund A, Möller T, Perfekt R (2002). Carcinoma of the parotid and submandibular glands – a study of survival in 2465 patients. Oral Oncol..

[21] Leverstein H, van der Wal JE, Tiwari RM, van der Waal I, Snow GB (1997). Surgical management of 246 previously untreated pleomorphic adenomas of the parotid gland. Br J Surg..

[22] McGurk M, Thomas BL, Renehan AG (2003). Extracapsular dissection for clinically benign parotid lumps: reduced morbidity without oncological compromise. Br J Cancer..

[23] Guntinas-Lichius O, Kick C, Klussmann JP, Jungehuelsing M, Stennert E (2004). Pleomorphic adenoma of the parotid gland: a 13-year experience of consequent management by lateral or total parotidectomy. Eur Arch Otorhinolaryngol..

[24] Carew JF, Spiro RH, Singh B, Shah JP (1999). Treatment of recurrent pleomorphic adenomas of the parotid gland. Otol Head Neck Surg..

[25] Spiro IJ, Wang CC, Montgomery WW (1993). Carcinoma of the parotid gland. Analysis of treatment results and patterns of failure after combined surgery and radiation therapy. Cancer..

